# Supplementary material for: Polymer hetero-electrolyte enabled solid-state 2.4-V Zn/Li hybrid batteries
Source: Nat Commun. 2024 May 3;15:3748. doi: 10.1038/s41467-024-47950-w (PMC11068732; doi:10.1038/s41467-024-47950-w)
Supplement: Supplementary file 1 — Supplementary Information [file 41467_2024_47950_MOESM1_ESM.pdf]

# Supplementary information

## Polymer hetero-electrolyte enabled solid-state 2.4-V Zn/Li hybrid batteries

Ze Chen,[1] Tairan Wang,[1] Zhuoxi Wu,[1] Yue Hou,[1] Ao Chen,[1] Yanbo Wang,[1] Zhaodong Huang,[1] Oliver G. Schmidt,[2,3] Minshen Zhu,*[2,3] Jun Fan,*[1] Chunyi Zhi*[1,4,5]

[1]Department of Materials Science and Engineering, City University of Hong Kong, 83 Tat Chee Avenue, Kowloon, Hong Kong 999077, China.

[2]Center for Materials, Architectures, and Integration of Nanomembranes (MAIN), TU Chemnitz, 09126 Chemnitz, Germany.

[3]Material Systems for Nanoelectronics, TU Chemnitz, 09107, Chemnitz, Germany, TU Chemnitz, 09126 Chemnitz, Germany.

[4]Hong Kong Center for Cerebro-Cardiovascular Health Engineering (COCHE), Shatin, NT, HKSAR, China.

[5]Hong Kong Institute for Clean Energy, City University of Hong Kong, Kowloon 999077, Hong Kong.

These authors contributed equally: Ze Chen, Tairan Wang.

E-mail: minshen.zhu@main.tu-chemnitz.de; junfan@cityu.edu.hk; cy.zhi@cityu.edu.hk

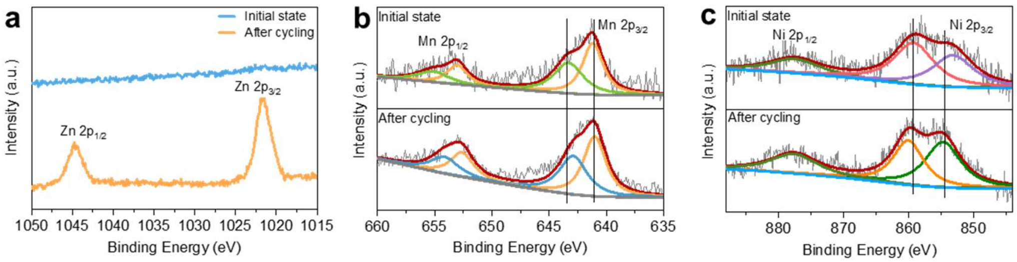


**Supplementary Fig. 1.** XPS spectra of the electrodes at the initial state and after cycling: a) Zn 2p, b) Mn 2p and c) Ni 2p.

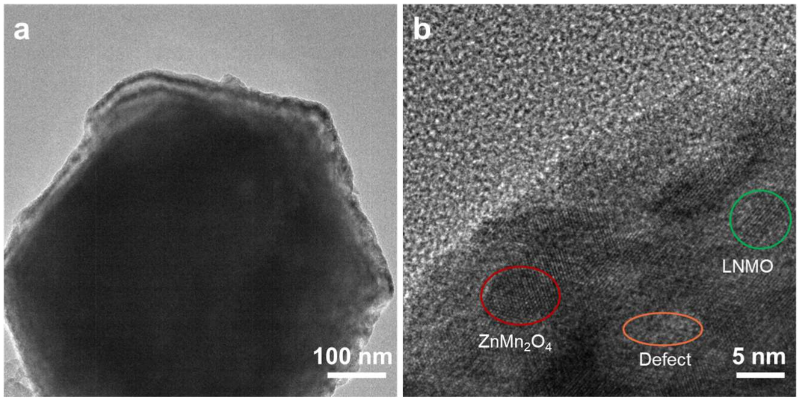


**Supplementary Fig. 2.** Morphologies of LNMO after cycling: a) and b) HRTEM images with different magnification of the LNMO electrode after cycling.

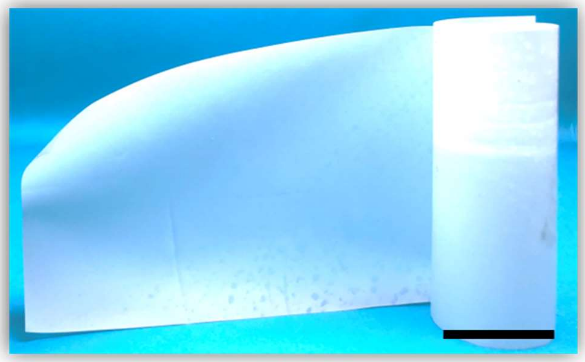

**Supplementary Fig. 3**. Optical picture of the fabricated PHE (scale bar: 1 cm).

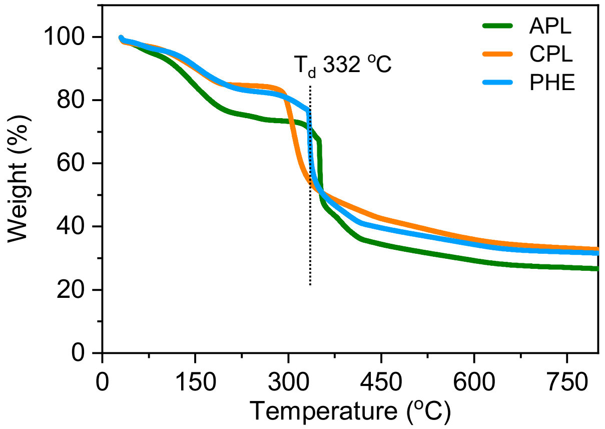


**Supplementary Fig. 4**. TGA curves of the as-prepared polymer electrolytes.

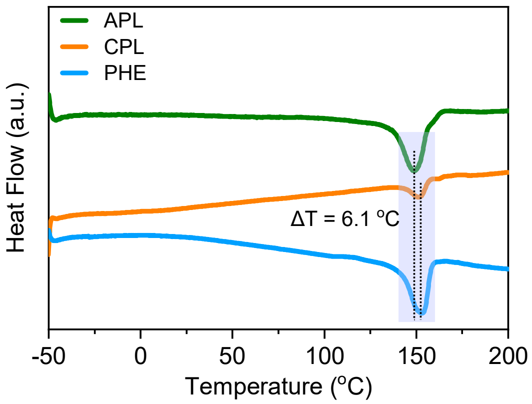


**Supplementary Fig. 5**. DSC curves of the as-prepared polymer electrolytes.

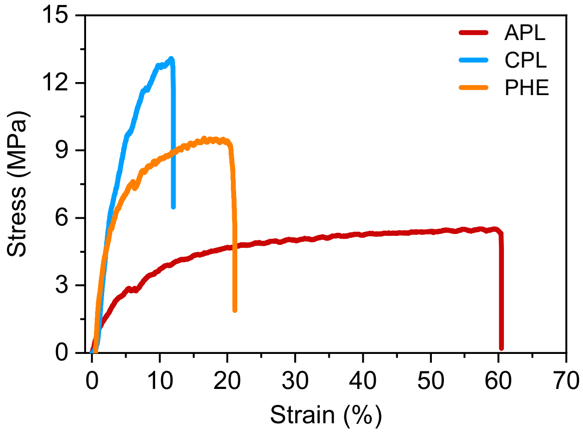


**Supplementary Fig. 6**. Strain-stress curves of the as-prepared polymer electrolytes.

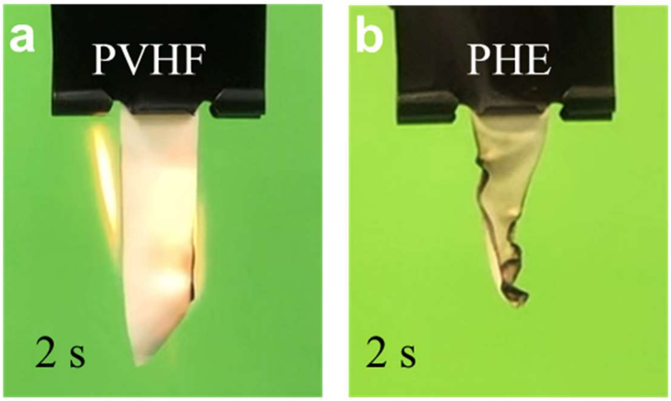


**Supplementary Fig. 7**. Flame test of the a) PVHF and b) PHE.

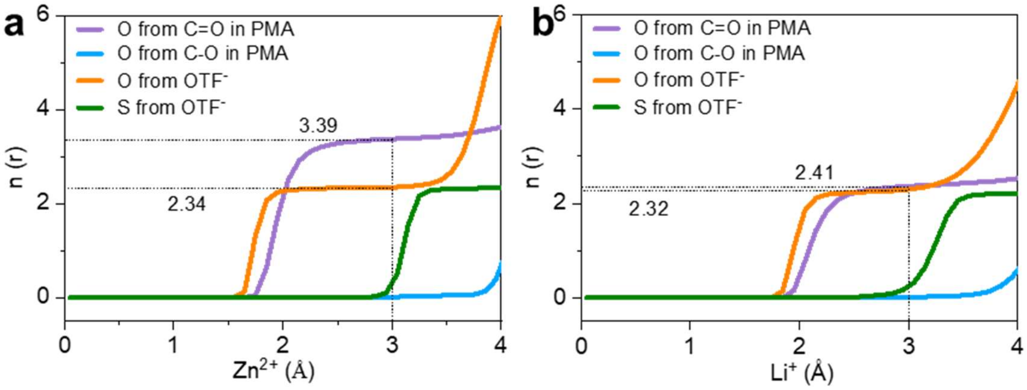


**Supplementary Fig. 8**. Coordination number, n(r) of a) $Zn(OTf)_2$ and b) LiOTf in PMA.

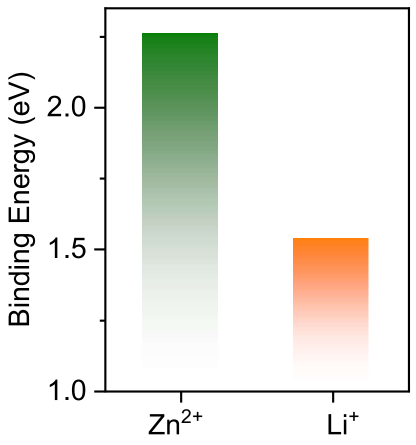


**Supplementary Fig. 9**. Binding energy of the $Zn^{2+}$ ion pairs with PMA and $Li^{+}$ ion pairs with PMA.

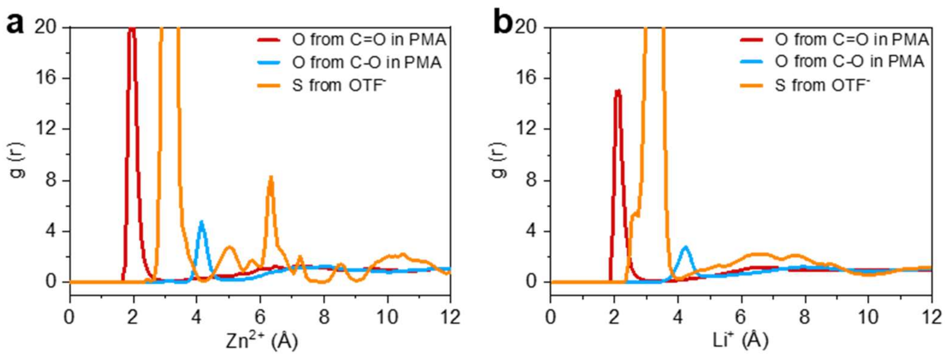


**Supplementary Fig. 10**. Radial Distribution Function (RDF) of a) $Zn^{2+}$ and b) $Li^{+}$ with PMA matrix.

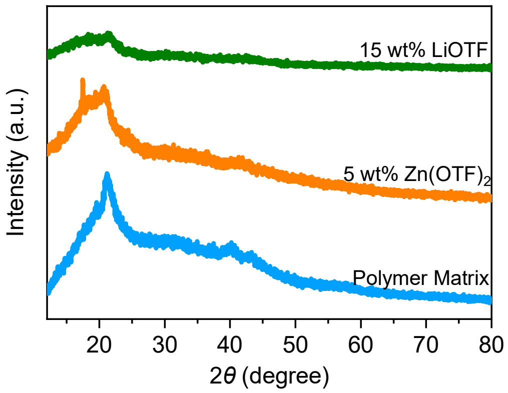


**Supplementary Fig. 11**. XRD patterns of $Zn(OTF)_2$ and LiOTF in the PMA based polymer matrix.

Obvious diffraction peaks of $Zn(OTF)_2$ can be observed even at the low Zn salt content (5 wt%), indicating the poor compatibility between the $Zn(OTF)_2$ and PMA. It is believed that PMA can effectively suppress the $Zn^{2+}$ migration in the CPL.

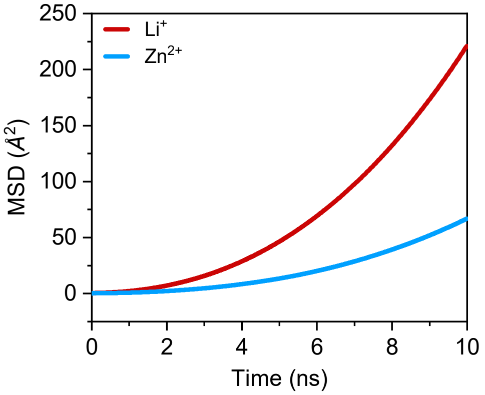


**Supplementary Fig. 12**. Mean square displacement (MSD) of $Zn^{2+}$ and $Li^{+}$ in PMA matrix.

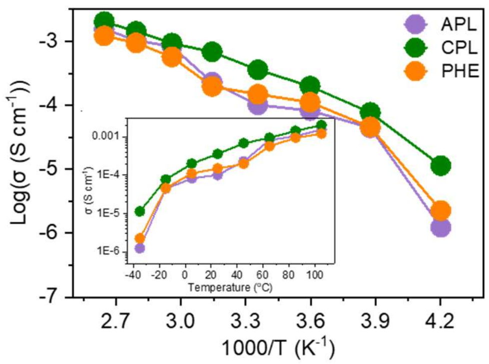


**Supplementary Fig. 13**. Ionic conductivities of the as-prepared APL, CPL and PHE under different temperatures based on Ti‖Ti cell (inset picture: variation of ionic conductivities with temperature changing).

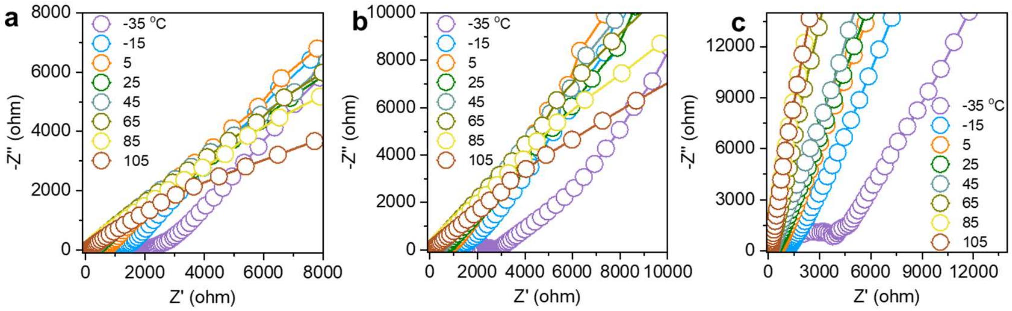


**Supplementary Fig. 14**. EIS curves of ionic conductivity test of a) APL; b) CPL and c) PHE.

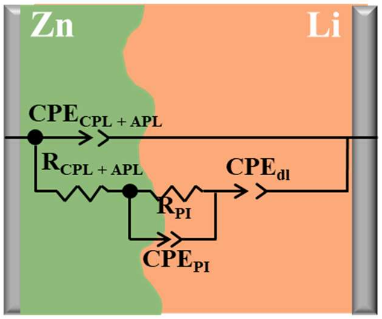


**Supplementary Fig. 15**. The correspondent equivalent circuits used to model its impedance-frequency response (CPE: constant phase element; $CPE_{dl}$: additional CPE element).

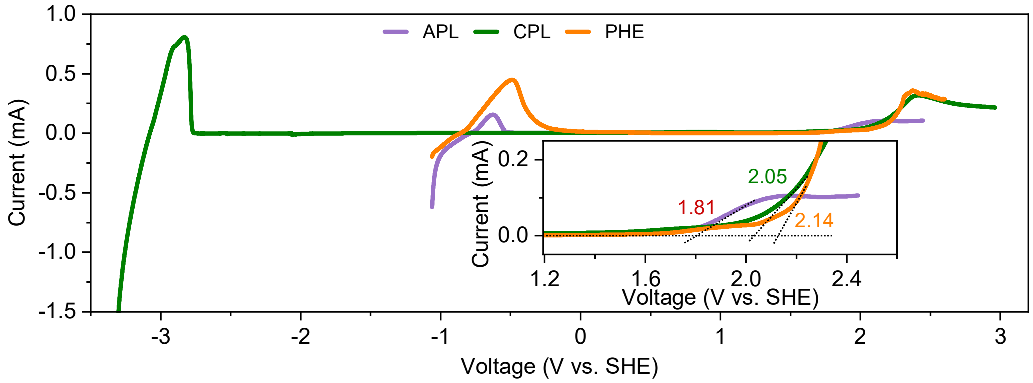


**Supplementary Fig. 16**. Linear sweep voltammetry (LSV) curves of the Zn or Li‖Ti cell based on the as-prepared SPEs (scan rate 0.5 mV $s^{-1}$) (inset picture: the selected area of the LSV plots for clearly identifying the decomposition potential of the SPEs).

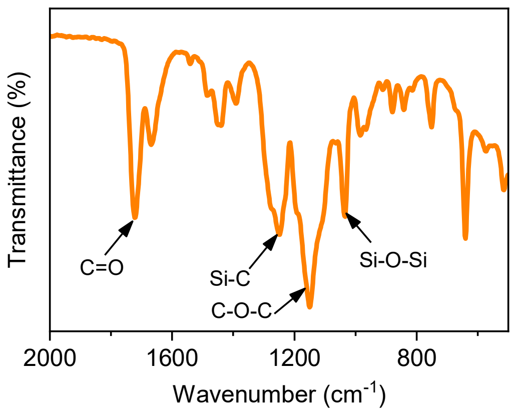


**Supplementary Fig. 17**. FT-IR spectra of the CPL with M-POSS-PMA crosslinking networks.

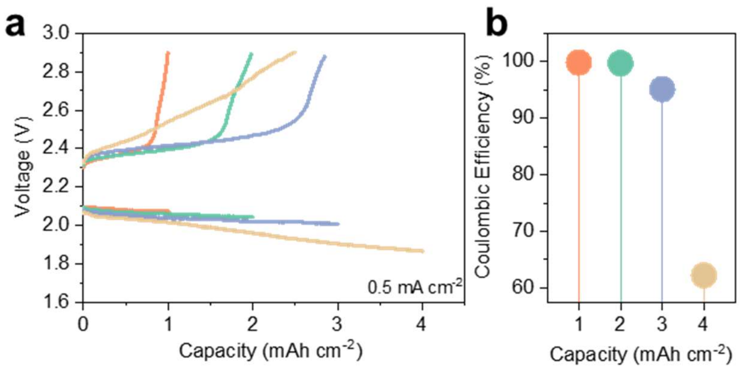


**Supplementary Fig. 18.** The platting and stripping performance of Zn based on the PHE: a) The galvanostatic charge/discharge (GCD) curves of the Li‖Cu cells at different capacities and b) the corresponding CE of the cells.

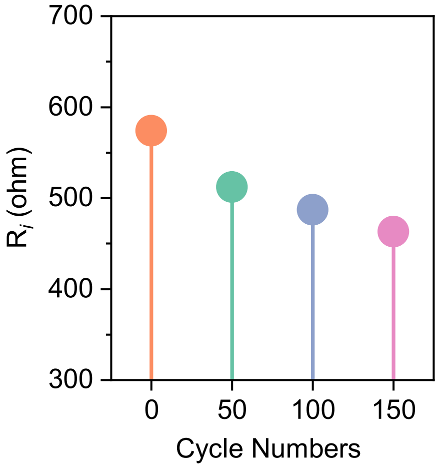


**Supplementary Fig. 19**. The interface resistance ($R_i$) of the Li‖Zn cell during cycling.

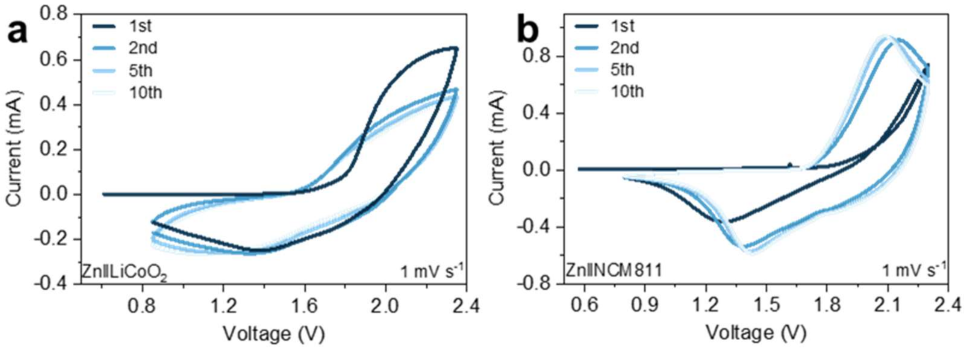


**Supplementary Fig. 20**. CV curves of a) Zn‖$LiCoO_2$ battery and b) Zn‖NCM 811 battery.

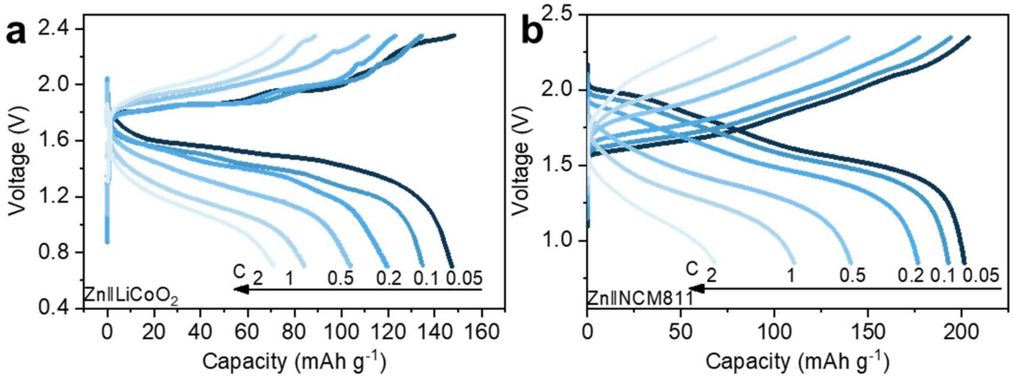


**Supplementary Fig. 21**. GCD curves of a) Zn‖$LiCoO_2$ battery and b) Zn‖NCM 811 battery.

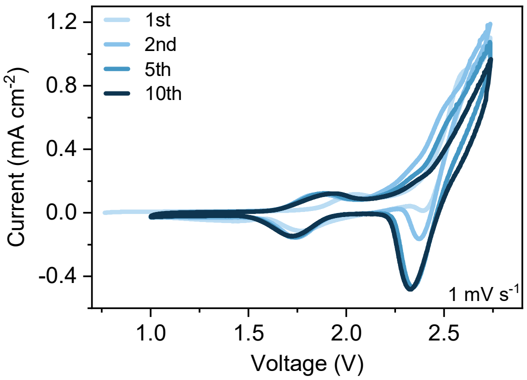


**Supplementary Fig. 22**. CV curves of the Zn‖LNMO pouch cell at 1 mV $s^{-1}$.

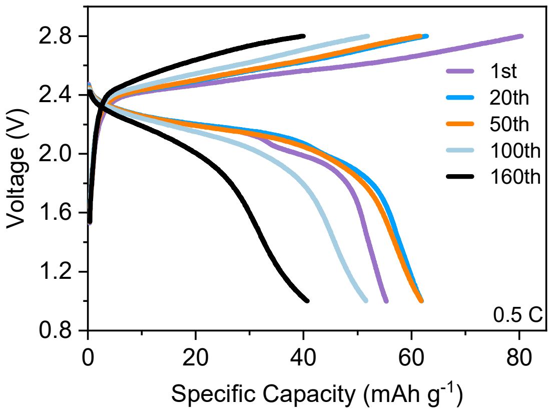


**Supplementary Fig. 23**. GCD curves of the solid Zn‖LNMO pouch cell at 0.5 C.

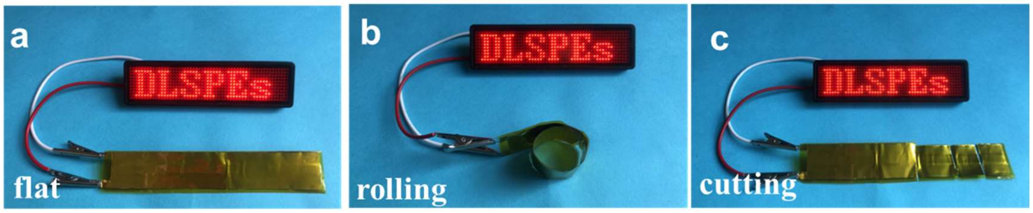


**Supplementary Fig. 24**. Zn‖LNMO pouch cell as the power supply of the LED display device: a) flat state, b) rolling state and c) cutting state.

**Supplementary Table 1**. Comparison of the Zn hybrid batteries in different systems.

| Cathodes | Electrolyte[a] | Capacity(mAh g$^{-1}$)@Rate | Energy Density (Wh Kg$^{-1}$) | Coulombic Efficiency (%) | Cycling life[b] | Ref |
|---|---|---|---|---|---|---|
| $MgMn_2O_4$ | 1M $MgSO_4$ + 1M $ZnSO_4$ | 125(0.1 A g$^{-1}$) | 150 | 99.0 | 80% (500)@0.5 A g$^{-1}$ | 1 |
| $LiV_3O_8$ | 0.5M LiOTF + 3M $Zn(OTf)_2$ | 380(0.1 A g$^{-1}$) | 285 | 88.8 | 87% (4000)@5 A g$^{-1}$ | 2 |
| $LiCoO_2$ | 1M $Zn(OAc)_2$ + 4M LiOAc + $NH_3H_2O$ | 91(0.5 C) | 173 | 98.7 | 95% (300)@2 C | 3 |
| FeHCF | 3M KOTf + 3M $Zn(OTf)_2$ | 150(0.5 A g$^{-1}$) | 165 | 94.0 | 57% (1000)@1 A g$^{-1}$ | 4 |
| $LiVPO_4F$ | 21 M LiTFSI + 2M $Zn(OTf)_2$ | 125(0.1 A g$^{-1}$) | 237 | 95.0 | 87% (600)@1 A g$^{-1}$ | 5 |
| $LiFePO_4$ | 1M LiOTf + 1M $Zn(OTf)_2$ | 158(0.5 C) | 174 | 94.0 | 89% (100)@ 1 C | 6 |
| $Na_3V_2(PO_4)_3$ | 1M $Ca(OTf)_2$ + 1M $Zn(OTf)_2$ | 81(1 C) | 105 | 99.0 | 74% (1300)@20 C | 7 |
| $LiMn_2O_4$ | 1m Zn(TFSI) + 20m LiTFSI | 65(0.2 C) | 112 | 99.9 | 85% (4000)@4 C | 8 |
| Ag | 0.1M $ZnCl_2$ | 104(1.5 A g$^{-1}$) | 99 | 99.0 | 93% (1300)@1 A g$^{-1}$ | 9 |
| Graphite | 3m $Zn(TFSI)_2$ in ethyl methyl carbonate | 110(0.1 A g$^{-1}$) | 231 | 94.0 | 96% (50)@0.1 A g$^{-1}$ | 10 |
| $Bi_2O_3$ | 6M KOH + 0.3M $Zn(OAc)_2$ | 323(0.3 C) | 245 | 98.2 | 49% (1000)@10 A g$^{-1}$ | 11 |
| $LNi_{0.5}Mn_{1.5}O_4$ | PHE | 113(0.05 C) | 289 | 99.6 | 77.3% (450)@0.5 C | This Work |

a. All the electrolytes are aqueous except special illustration.

b. The percentage means capacity retention, and the number in brackets means cycle numbers.

**Supplementary Table 2**. The calculation process of energy density of the Zn‖LNMO batteries.

| $V$(Average voltage) | $C_s$(specific capacity) | $m_A$(loading mass of active materials) | $V_T$(volume of batteries based on the anode, cathode and PHE) | $V_T$(volume of the cell) |
|---|---|---|---|---|
| 2.35 V | 63 mAh $g^{-1}$ | 0.65 g | 0.68 $cm^{-3}$ | 1.78 $cm^{-3}$ |
| $E_V = \frac{V*C_S*m_A}{V_T}$, 142 Wh $L^{-1}$(based on the anode, cathode and PHE) and 54 Wh $L^{-1}$(based on the cell) | | | | |

## References


1. Soundharrajan V*, et al.* Aqueous Magnesium Zinc Hybrid Battery: An Advanced High-Voltage and High-Energy MgMn2O4 Cathode. *ACS Energy Letters* **3**, 1998-2004 (2018).

2. Pang Q*, et al.* High-Capacity and Long-Lifespan Aqueous LiV3O8/Zn Battery Using Zn/Li Hybrid Electrolyte. *Nanomaterials* **11**, 1429 (2021).

3. Yu Z, Cao L, Liu H, Wang D-W. High voltage aqueous Zn/LiCoO2 hybrid battery under mildly alkaline conditions. *Energy Storage Materials* **43**, 158-164 (2021).

4. Ni G, Hao Z, Zou GY, Cao FH, Qin L, Zhou CG. High-Performance Aqueous Rechargeable K/Zn Hybrid Batteries Based on Berlin Green Cathode Materials. *ChemElectroChem* **9**, e202101351 (2022).

5. Liu ZX*, et al.* A Flexible Solid-State Aqueous Zinc Hybrid Battery with Flat and High-Voltage Discharge Plateau. *Advanced Energy Materials* **9**, 1902473 (2019).

6. Hao J*, et al.* Toward High-Performance Hybrid Zn-Based Batteries via Deeply Understanding Their Mechanism and Using Electrolyte Additive. *Advanced Functional Materials* **29**, 1903605 (2019).

7. Zhao S*, et al.* An advanced Ca/Zn hybrid battery enabled by the dendrite-free zinc anode and a reversible calcification/decalcification NASICON cathode. *Science Bulletin* **68**, 56-64 (2023).

8. Wang F*, et al.* Highly reversible zinc metal anode for aqueous batteries. *Nature Materials* **17**, 543-549 (2018).

9. Liang GJ*, et al.* Commencing mild Ag–Zn batteries with long-term stability and ultra-flat voltage platform. *Energy Storage Materials* **25**, 86-92 (2020).

10. Wang G*, et al.* An Anode-Free Zn–Graphite Battery. *Advanced Materials* **34**, 2201957 (2022).

11. Wang D*, et al.* A zinc battery with ultra-flat discharge plateau through phase transition mechanism. *Nano Energy* **71**, 104583 (2020).
